# Predictive Factors for the Formation of Viable Embryos in Subfertile Patients with Diminished Ovarian Reserve: A Clinical Prediction Study

**DOI:** 10.1007/s43032-024-01469-z

**Published:** 2024-02-26

**Authors:** Keng Feng, Zhao Zhang, Ling Wu, Lingling Zhu, Xiang Li, Derong Li, Luhai Ruan, Yudi Luo

**Affiliations:** 1 Center of Reproductive Medicine, Yulin Maternal and Child Health Hospital, Yulin, China; 2 Center of Reproductive Medicine, Qinzhou Maternal and Child Health Hospital, Qinzhou, China; 3 Pediatric Surgery, The People’s Hospital of Guangxi Zhuang Autonomous Region, Nanning, China

**Keywords:** Diminished ovarian reserve, Prediction model, Usable blastocyst, Nomogram

## Abstract

This study aims to construct and validate a nomogram for predicting blastocyst formation in patients with diminished ovarian reserve (DOR) during in vitro fertilization (IVF) procedures. A retrospective analysis was conducted on 445 DOR patients who underwent in vitro fertilization (IVF)/intracytoplasmic sperm injection (ICSI) at the Reproductive Center of Yulin Maternal and Child Health Hospital from January 2019 to January 2023. A total of 1016 embryos were cultured for blastocyst formation, of which 487 were usable blastocysts and 529 did not form usable blastocysts. The embryos were randomly divided into a training set (711 embryos) and a validation set (305 embryos). Relevant factors were initially identified through univariate logistic regression analysis based on the training set, followed by multivariate logistic regression analysis to establish a nomogram model. The prediction model was then calibrated and validated. Multivariate stepwise forward logistic regression analysis showed that female age, normal fertilization status, embryo grade on D2, and embryo grade on D3 were independent predictors of blastocyst formation in DOR patients. The Hosmer–Lemeshow test indicated no statistical difference between the predicted probabilities of blastocyst formation and actual blastocyst formation (*P* > 0.05). These results suggest that female age, normal fertilization status, embryo grade on D2, and embryo grade on D3 are independent predictors of blastocyst formation in DOR patients. The clinical prediction nomogram constructed from these factors has good predictive value and clinical utility and can provide a basis for clinical prognosis, intervention, and the formulation of individualized medical plans.

## Introduction

Infertility affects approximately 10–15% of couples worldwide, with female factors accounting for approximately 40% of all cases [1]. One of the common causes of female infertility is diminished ovarian reserve (DOR), also known as premature ovarian insufficiency or ovarian function decline, which refers to a reduction in the quantity or quality of oocytes in the ovaries [2]. DOR refers to the decline in the ability of the ovaries to produce oocytes and the reduction in oocyte quality in women under the age of 40 due to various reasons, leading to a decrease in fertility and a deficiency in sex hormones. According to the Bologna criteria, DOR is defined as having low levels of anti-Müllerian hormone (AMH < 0.5–1.1 ng/ml), a low antral follicle count (AFC < 5–7), and/or elevated baseline follicle-stimulating hormone (FSH) levels in women of reproductive age [3]. Potential causes of DOR mainly include autoimmune diseases, hereditary chromosomal and genetic disorders, environmental hazards, and iatrogenic factors. Meanwhile, Park SU et al. have elaborated on the molecular mechanisms associated with the onset of DOR, considering gene mutations and errors in meiotic recombination, as well as related factors such as DNA damage, telomere changes, reactive oxygen species, and mitochondrial dysfunction [4]. How to achieve clinical pregnancy in DOR patients is a challenge for assisted reproductive technology, with an incidence rate of 10–30% [5]. Among the infertile population, there is a trend of increasing incidence and younger age in recent years. In recent years, with the improvement in sequential embryo culture techniques, blastocyst culture technology has become more mature. Blastocyst culture undergoes a developmental process involving cell fusion, formation, and expansion of the blastocoel, eliminating some embryos with poor developmental potential. Additionally, studies by Papanikolaou EG reported that blastocyst transfer is more conducive to increasing the synchrony between endometrial and embryo development, improving clinical pregnancy rates and birth rates while also reducing the risk of multiple pregnancies [6]. However, there is a risk of culture failure in blastocyst culture, and various studies report that the rate of blastocyst formation is 40–60% in different age groups and using different culture methods [7, 8]. Therefore, in clinical practice, some patients may experience the situation of having no transferable blastocysts, and the risk of having no usable blastocysts after sequential culture of embryos is higher in DOR patients, causing severe economic and psychological burdens.

Previous studies have explored the predictive factors for embryo formation in infertile populations, but limited research has specifically addressed this issue in DOR patients, such as Mi Z et al. suggesting that D2 cleavage-stage embryos with four cells have the highest rate of blastocyst formation [9]; Bassil R et al. considering the diameter of the oocyte as an important factor affecting blastocyst formation, with embryos formed from oocytes measuring between 105.96 and 118.69 μm in diameter having the highest probability of forming high-quality D5 blastocysts [10]; and Yang SH et al. that used time-lapse imaging systems to observe embryo morphokinetics and morphokinetic parameters, finding that the time of pronuclear fading after fertilization and the timing of blastomere division and abnormal division patterns are key factors affecting blastocyst formation [11]. However, there is a lack of research reports on the prediction of blastocyst formation combining clinical data such as patient age and endocrine status with embryo morphokinetic parameters. In this study, we aim to conduct a clinical prediction study to investigate the factors associated with the formation of usable embryos in DOR patients undergoing ART procedures.

## Materials and Methods

### Patient Data

A retrospective analysis was conducted on 445 patients with diminished ovarian reserve (DOR) who underwent in vitro fertilization/intracytoplasmic sperm injection (IVF/ICSI) at the Reproductive Center of Yulin Maternal and Child Health Hospital from January 2019 to January 2023, involving a total of 1016 embryos for blastocyst culture. The inclusion criteria are as follows: (1) antral follicle count (AFC) < 6 in both ovaries, serum anti-Müllerian hormone (AMH) levels < 0.5–1.1 ng/ml, or baseline follicle-stimulating hormone (FSH) levels ≥ 10 IU/L for two consecutive menstrual cycles; (2) patients undergoing IVF/ICSI and blastocyst culture; and (3) patients fully informed about the IVF embryo transfer process and who have signed an informed consent form. The exclusion criteria are as follows: (1) patients receiving reproductive assistance through ICSI with testicular sperm aspiration (TESA) or round spermatid injection (ROSI); (2) those without oocyte retrieval on the day of oocyte pick-up, complete fertilization failure, or with no cleavage embryos available for blastocyst culture on day 3; (3) either partner suffering from severe psychiatric disorders, acute urinary or genital infections or sexually transmitted diseases, or hereditary diseases that are deemed inappropriate for procreation under the “Maternal and Infant Health Care Law” of the People’s Republic of China, and for which prenatal diagnosis or preimplantation genetic diagnosis is currently unfeasible. The study was conducted following the approval of the Medical Ethics Committee of Yulin Maternal and Child Health Hospital, Guangxi.

### Data Collection

Key observation indicators were collected from the hospital’s reproductive medical record management system, encompassing complete clinical and laboratory data for both male and female partners from January 2019 to January 2023. The following demographic and clinical data were obtained: maternal age, paternal age, infertility duration, infertility type, number of blastocysts cultured, normal fertilization, the blastomere number of D2, embryo fragmentation of D2, day 3 embryo grade, fusion embryos, the blastomere number of D3, embryo fragmentation of D3, day 2 embryo grade, ovarian stimulation protocol, type of fertilization, maternal BMI, basal FSH, basal LH, basal PRL, basal E2, basal T, basal P, AMH, AFC, total Gn dosage, duration of stimulation, initial FSH dosage, serum E2 level on the HCG trigger day, serum LH level on the HCG trigger day, serum P level on the HCG trigger day, volume of semen after treatment, semen density after treatment, and semen recovery rate. The data of outcome, usable blastocyst, were also collected.

### Statistical Analysis

The dataset collected was randomly divided into training and validation cohorts at a ratio of 7:3, and the variables were compared. Non-normal data were presented as median (interquartile ranges). In the univariate analysis, chi-square test or Fisher’s exact test was used to analyze the categorical variables, while the Student’s* t*-test or rank-sum test was used to examine the continuous variables. In the training cohort, the least absolute shrinkage and selection operator (LASSO) logistic regression analysis was used for multivariate analysis to screen the independent risk factors and build a prediction nomogram for usable blastocyst. The performance of the nomogram was assessed using the receiver operating characteristic (ROC) curve and calibration curve, with the area under the ROC curve (AUC) ranging from 0.5 (no discriminant) to 1 (complete discriminant). A decision curve analysis (DCA) was also performed to determine the net benefit threshold of prediction. Results with a *p*-value of < 0.05 were considered significant. All statistical analyses were performed using the R software (version 4.2.2).

## Results

### Patient Characteristics

#### General Characteristics

This retrospective study included records of 1016 blastocyst cultures, which were randomly divided into a training set and a validation set at a ratio of 7:3. The baseline demographic and clinical characteristics of the study population are summarized in Table 1. The characteristics include maternal age, paternal age, infertility duration, infertility type, number of blastocysts cultured, normal fertilization, the blastomere number of D2, embryo fragmentation of D2, day 3 embryo grade, fusion embryos, the blastomere number of D3, embryo fragmentation of D3, day 2 embryo grade, ovarian stimulation protocol, type of fertilization, maternal BMI, basal FSH, basal LH, basal PRL, basal E2, basal T, basal P, AMH, AFC, total Gn dosage, duration of stimulation, initial FSH dosage, serum E2 level on the HCG trigger day, serum LH level on the HCG trigger day, serum P level on the HCG trigger day, volume of semen after treatment, semen density after treatment, semen recovery rate, and semen recovery rate. Overall, the baseline characteristics were generally well-balanced between the training cohort and the internal test cohort, with non-significant *p*-values for most comparisons, suggesting that the two cohorts were suitable for predictive research.

**Table 1:** Patient demographics and baseline characteristics

Characteristic	Cohort		*p*-value^2^
	Training cohort, *N* = 725^1^	Internal test cohort, *N* = 310^1^	
Maternal age			0.625
Median (IQR)	39.0 (35.0, 41.0)	39.0 (35.0, 41.0)	
Paternal age			0.286
Median (IQR)	40.0 (35.0, 43.0)	39.4 (35.0, 42.0)	
Infertility duration			0.159
Median (IQR)	5.0 (3.0, 9.0)	5.0 (3.0, 8.0)	
Infertility type			0.542
Primary infertility	131 (18.1%)	61 (19.7%)	
Secondary infertility	594 (81.9%)	249 (80.3%)	
Number of blastocysts cultured			0.280
Median (IQR)	3.00 (2.00, 4.00)	2.00 (2.00, 4.00)	
Normal fertilization			0.243
No	114 (15.7%)	40 (12.9%)	
Yes	611 (84.3%)	270 (87.1%)	
The blastomere number of D2			0.239
Median (IQR)	4.00 (4.00, 4.00)	4.00 (4.00, 4.00)	
Embryo fragmentation of D2			0.376
Median (IQR)	0.0 (0.0, 5.0)	0.0 (0.0, 5.0)	
Day 3 embryo grade			0.636
I	390 (53.8%)	166 (53.5%)	
II	174 (24.0%)	68 (21.9%)	
III	161 (22.2%)	76 (24.5%)	
Fusion embryos			0.323
No	683 (94.2%)	287 (92.6%)	
Yes	42 (5.8%)	23 (7.4%)	
The blastomere number of D3			0.064
Median (IQR)	8.00 (7.00, 8.61)	8.00 (7.00, 8.98)	
Embryo fragmentation of D3			0.708
Median (IQR)	0.0 (0.0, 10.0)	0.0 (0.0, 10.0)	
Day 2 embryo grade			0.878
I	219 (30.2%)	89 (28.7%)	
II	123 (17.0%)	55 (17.7%)	
III	383 (52.8%)	166 (53.5%)	
Ovarian stimulation protocol			0.221
Median (IQR)	1.00 (1.00, 2.00)	1.00 (1.00, 2.00)	
Type of fertilization			0.753
IVF	540 (74.5%)	228 (73.5%)	
ICSI	185 (25.5%)	82 (26.5%)	
Maternal BMI			0.306
Median (IQR)	22.76 (20.81, 24.65)	22.86 (20.86, 24.97)	
Basal FSH			0.617
Median (IQR)	7.2 (5.8, 9.2)	6.9 (5.8, 9.3)	
Basal LH			0.930
Median (IQR)	2.60 (1.92, 3.49)	2.59 (1.92, 3.59)	
Basal PRL			0.119
Median (IQR)	15 (10, 20)	16 (11, 21)	
Basal E2			0.137
Median (IQR)	33 (23, 48)	30 (22, 45)	
Basal T			0.621
Median (IQR)	0.25 (0.20, 0.31)	0.25 (0.20, 0.30)	
Basal P			0.977
Median (IQR)	0.30 (0.20, 0.40)	0.30 (0.20, 0.40)	
AMH			0.087
Median (IQR)	0.68 (0.46, 0.88)	0.66 (0.43, 0.82)	
AFC			0.395
Median (IQR)	4.91 (4.00, 6.00)	4.96 (4.00, 6.00)	
Total Gndosage			0.343
Median (IQR)	2,175 (1,610, 2,700)	2,250 (1,735, 2,700)	
Duration of stimulation			0.169
Median (IQR)	9.00 (7.91, 10.00)	9.00 (8.00, 10.00)	
Initial FSH dosage			0.518
Median (IQR)	225 (225, 300)	225 (225, 300)	
Serum E2 level on the hCG trigger day			0.314
Median (IQR)	1,125 (743, 1,568)	1,093 (749, 1,577)	
Serum LH level on the hCG trigger day			0.913
Median (IQR)	1.78 (1.20, 2.91)	1.77 (1.16, 2.91)	
Serum P level on the hCG trigger day			0.675
Median (IQR)	0.30 (0.20, 0.50)	0.30 (0.20, 0.50)	
Volume of semen after treatment			0.539
Median (IQR)	0.50 (0.50, 0.50)	0.50 (0.50, 0.50)	
Semen density after treatment			0.277
Median (IQR)	4.00 (2.50, 5.00)	3.00 (2.00, 5.00)	
Semen recovery rate			0.298
Median (IQR)	5 (3, 9)	5 (3, 9)	

^1^*n* (%)

^2^Wilcoxon rank-sum test; Pearson’s chi-squared test

### Predictive Model

#### LASSO Regression Model

**Fig. 1:**
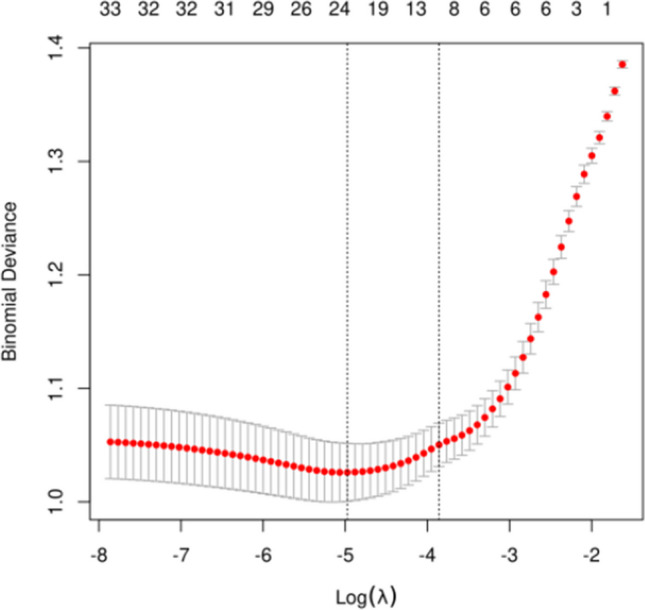
Lasso regression cross-validation plot

**Fig. 2:**
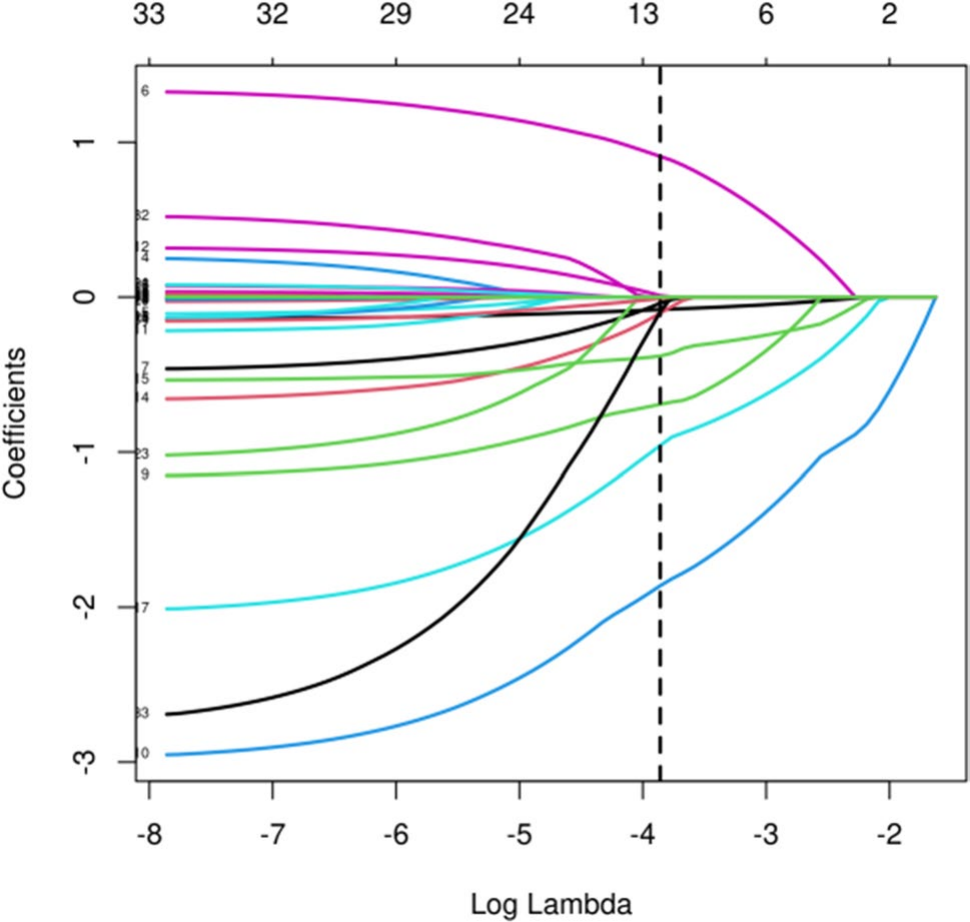
Lasso regression coefficient path plot

**Fig. 3:**
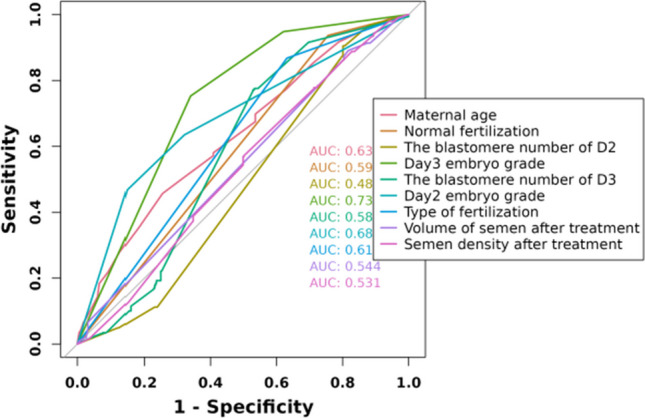
ROC curve analysis of 9 candidate diagnostic indicators

The candidate predictors, maternal age, paternal age, infertility duration, infertility type, number of blastocysts cultured, normal fertilization, the blastomere number of D2, embryo fragmentation of D2, day 3 embryo grade, fusion embryos, the blastomere number of D3, embryo fragmentation of D3, day 2 embryo grade, ovarian stimulation protocol, type of fertilization, maternal BMI, basal FSH, basal LH, basal PRL, basal E2, basal T, basal P, AMH, AFC, total Gn dosage, duration of stimulation, initial FSH dosage, serum E2 level on the HCG trigger day, serum LH level on the HCG trigger day, serum P level on the HCG trigger day, volume of semen after treatment, semen density after treatment, and semen recovery rate, were included in the original model, which were then reduced to 9 potential predictors using LASSO regression analysis performed in the training cohort. The coefficients are shown in the following table, and a coefficient profile is plotted in Fig. 1. A cross-validated error plot of the LASSO regression model is also shown in Fig. 2. The most regularized and parsimonious model, with a cross-validated error within one standard error of the minimum, included 9 variables. As shown in Fig. 3, the ROC analysis of the abovementioned variables yielded AUC values greater than 0.5.

#### Multivariate Logistic Analyses

Using multivariate logistic regression analysis, further analysis was conducted on the optimal matching factors identified by Lasso regression. The results revealed that five variables—female age, normal fertilization, day 2 cleavage-stage embryo grading, day 3 cleavage-stage embryo grading, and method of fertilization—were independent predictors of blastocyst formation in patients with diminished ovarian reserve (DOR), as shown in Table 2

**Table 2:** Results of multivariate logistic regression

Characteristic	*N*	Event *N*	OR^1^	95% CI^1^	*p*-value
Maternal age	725	348	0.88	0.84, 0.92	< 0.001
Normal fertilization					
No	114	22	—	—	
Yes	611	326	3.68	2.07, 6.76	< 0.001
The blastomere number of D2	725	348	0.63	0.48, 0.81	< 0.001
Day 3 embryo grade					
I	390	262	—	—	
II	174	68	0.40	0.24, 0.65	< 0.001
III	161	18	0.07	0.03, 0.14	< 0.001
The blastomere number of D3	725	348	0.31	0.12, 1.54	0.091
Day 2 embryo grade					
I	219	163	—	—	
II	123	58	0.53	0.28, 0.98	0.042
III	383	127	0.61	0.37, 1.02	0.061
Type of fertilization					
IVF	540	302	—	—	
ICSI	185	46	0.17	0.10, 0.29	< 0.001
Volume of semen after treatment	725	348	0.17	0.02, 1.16	0.073
Semen density after treatment	725	348	0.89	0.78, 1.02	0.108

^1^*OR* odds ratio, *CI* confidence interval

#### Nomogram Prediction Model

##### Construction of a Nomogram Predicting Usable Blastocyst Formation in DOR Patients

**Fig. 4:**
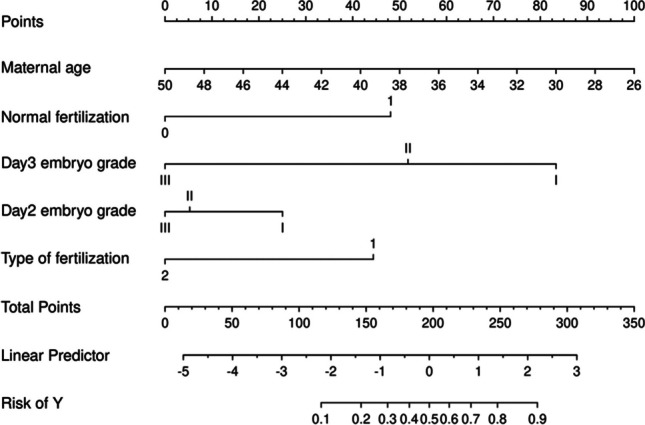
Nomogram prediction model. Normal fertilization: 0 represents non-2PN (pronuclear), and 1 represents 2PN (pronuclear). Type of fertilization: 1 represents in vitro fertilization (IVF), and 2 represents intracytoplasmic sperm injection (ICSI)

Based on the results of the multivariate logistic regression analysis, a nomogram was constructed to predict the formation of usable blastocysts in DOR patients, incorporating female age, normal fertilization, day 2 cleavage-stage embryo grading, day 3 cleavage-stage embryo grading, and method of fertilization. The model for this nomogram is shown in Fig. 4. A nomogram is a graphical calculating device, a two-dimensional diagram designed to allow the approximate graphical computation of a function. It is based on the principles of multivariable regression analysis and integrates multiple prognostic indicators by employing scaled lines drawn proportionally on the same plane to express the interrelationships among various variables in a predictive model. Each variable is represented by a line segment with marked scales, indicating the range of possible values for that variable, while the length of the line segment reflects the impact of that factor on the outcome event. As illustrated in Fig. 4, the age of patients with diminished ovarian reserve (DOR) is the most significant factor affecting the formation of usable blastocysts. This is followed in importance by day 3 embryo grade, normal fertilization, type of fertilization, and day 2 embryo grade.

#### ROC Curves of the Nomogram Prediction Model

##### Analysis of Calibration of the Nomogram for Predicting Usable Blastocyst Formation in DOR Patients

**Fig. 5:**
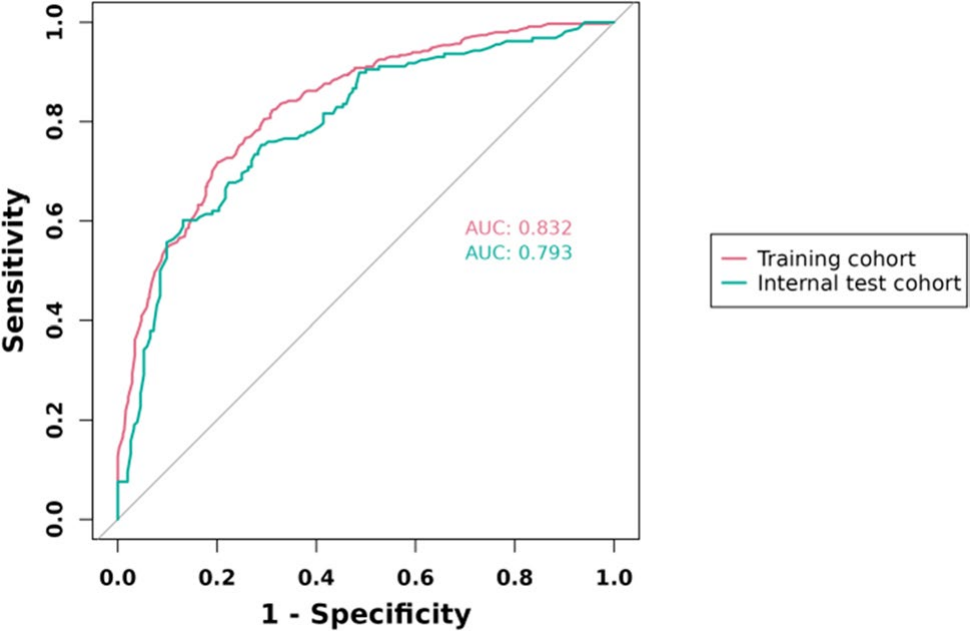
ROC curves of the nomogram prediction model

The area under the receiver operating characteristic (ROC) curve (AUC) for the model constructed from the training set was 0.832. Internal validation of the nomogram model was performed, and the AUC for the validation set was 0.793, as seen in Fig. 5. In the nomogram prediction model, the individual ROC for each of the five included factors was ≤ 0.63.The calibration plots of the nomogram in the different cohorts are plotted in the following figures, which demonstrate a good correlation between the observed and predicted Usable blastocyst. The results showed that the original nomogram was still valid for use in the validation sets, and the calibration curve of this model was relatively close to the ideal curve, which indicates that the predicted results were consistent with the actual findings.

#### Decision Curve Analysis

**Fig. 6:**
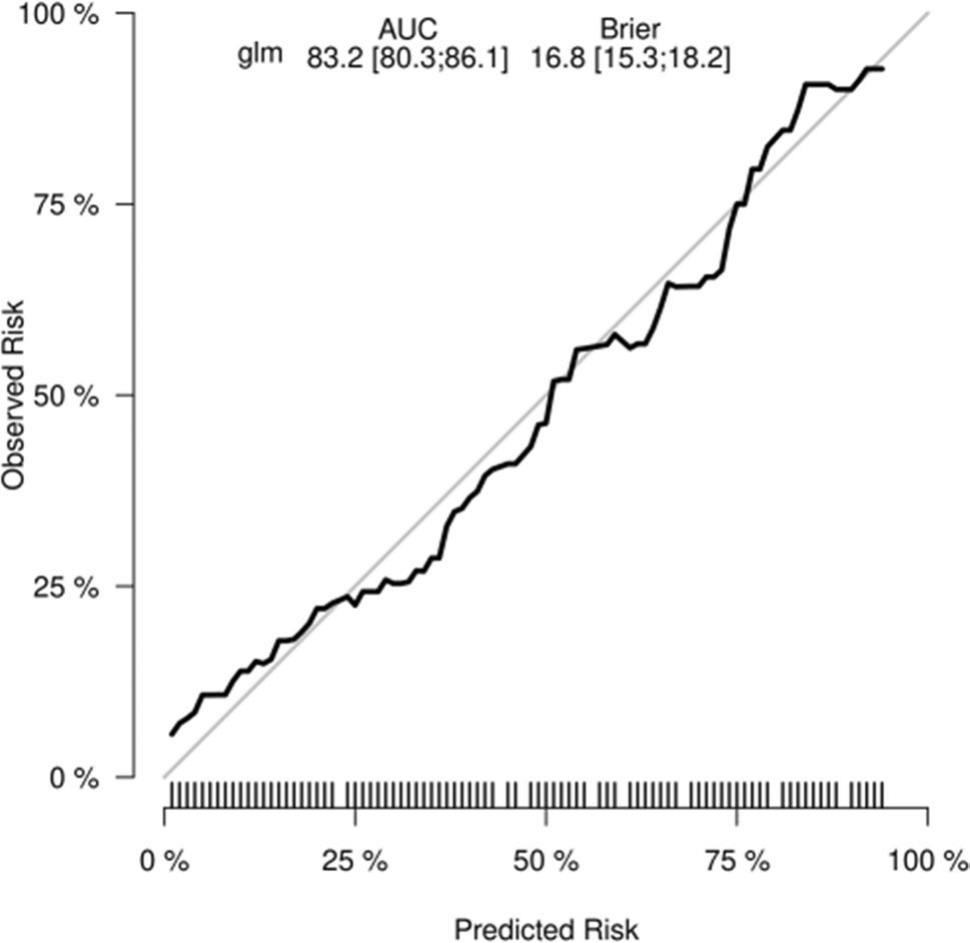
Calibration curve of the nomogram prediction mode for the training cohort

**Fig. 7:**
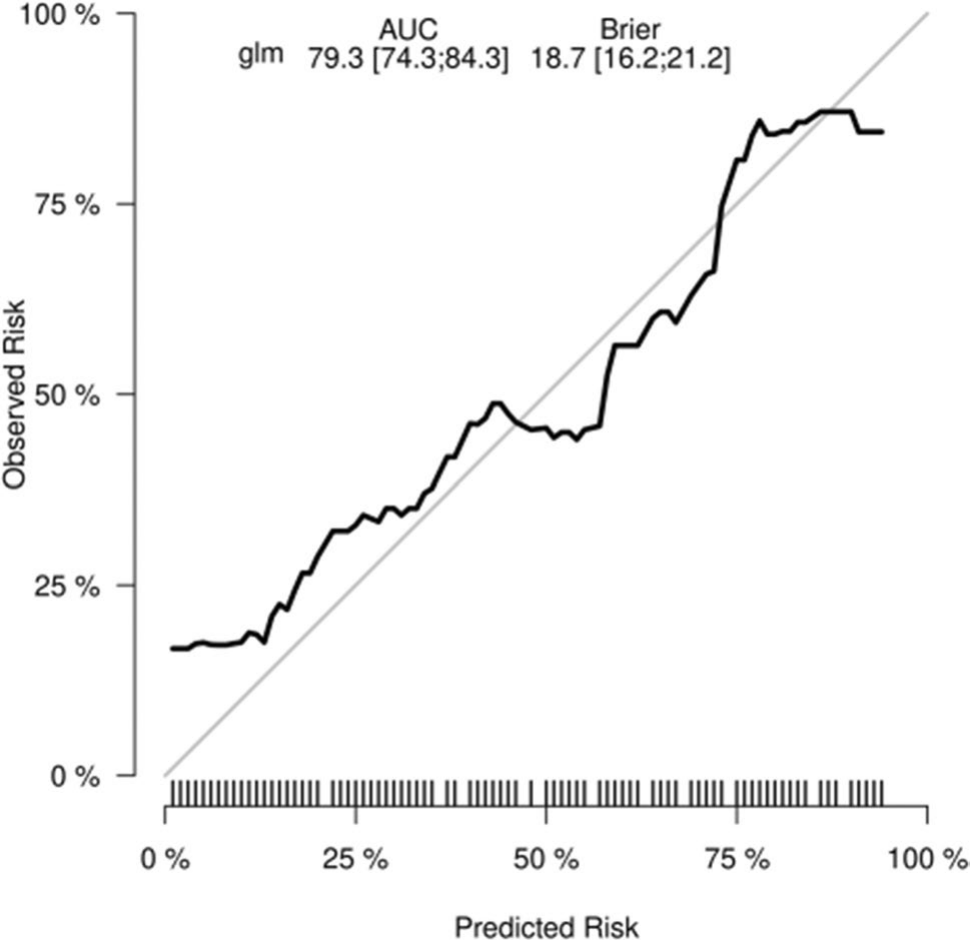
Calibration curve of the nomogram prediction mode for the internal test cohort

**Fig. 8:**
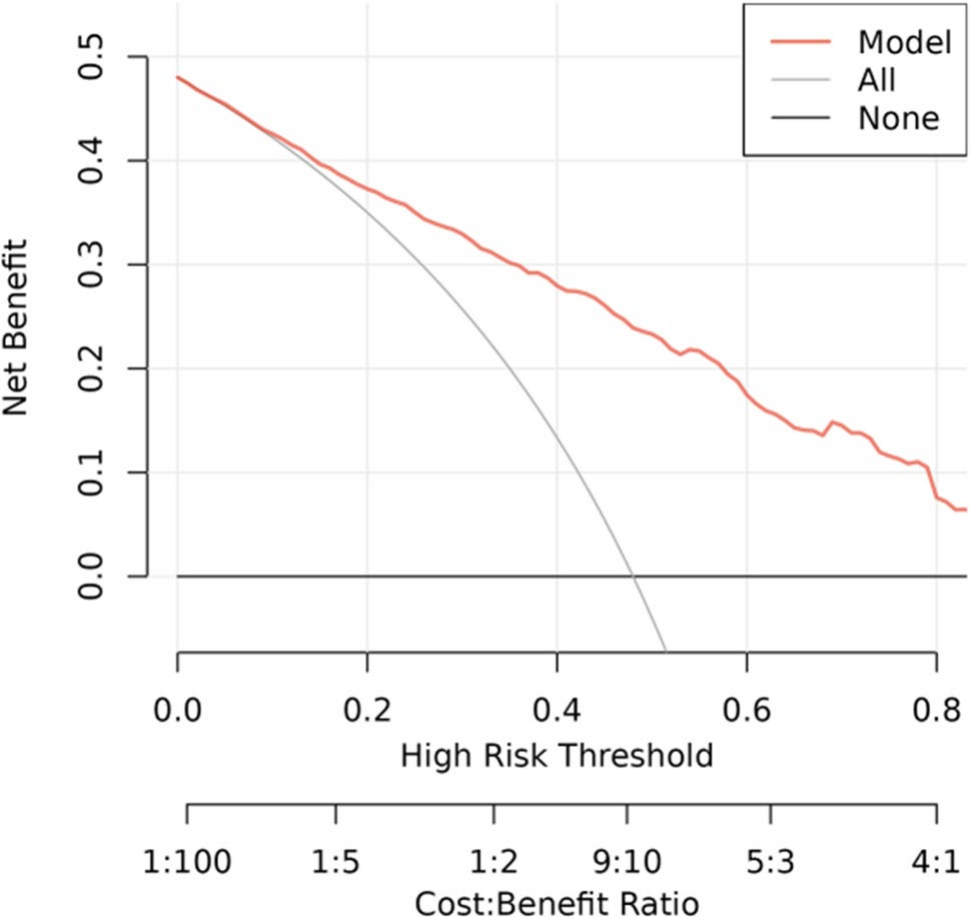
Decision curve analysis of the nomogram of the training cohort

**Fig. 9:**
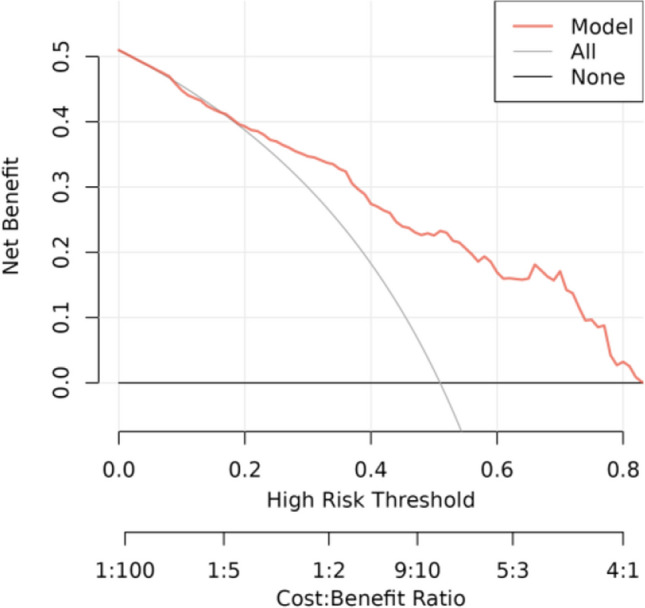
Decision curve analysis of the nomogram of the internal test cohort

The following figure displays the DCA curves related to the nomogram. A high-risk threshold probability indicates the chance of significant discrepancies in the model’s prediction when clinicians encounter major flaws while utilizing the nomogram for diagnostic and decision-making purposes. This research shows that the nomogram offers substantial net benefits for clinical application through its DCA curve (Figs. 6, 7, 8 and 9).

## Discussion

As reported by Awonuga AO et al., endometrial receptivity and embryo quality are key factors influencing embryo implantation. Despite numerous studies suggesting that enhancing endometrial blood flow or improving the intrauterine environment may increase live birth rates, the authors argue that this perspective lacks clear, high-quality evidence from systematic reviews and believe that improving embryo quality might be more effective than endometrial treatment [12]. Arab S et al. reported that in frozen-thawed embryo transfer, transferring two low-quality blastocysts does not increase clinical pregnancy rates but may raise the risk of multiple pregnancies; therefore, single blastocyst transfer is still recommended [13]. This underscores the importance of blastocyst culture in guiding assisted reproductive technology (ART) transfer strategies. However, patients with diminished ovarian reserve (DOR) have fewer available oocytes, hence a reduced number of viable embryos and a higher risk of not forming usable blastocysts after further culture. The absence of transferable embryos not only imposes an economic burden on DOR patients but also causes severe psychological stress. In this study, LASSO regression was utilized to identify factors affecting the formation of viable blastocysts in DOR patients. The results revealed nine factors: female age, cell count of day 2 cleavage-stage embryos, grade of day 2 cleavage-stage embryos, cell count of day 3 cleavage-stage embryos, grade of day 3 cleavage-stage embryos, normal fertilization, fertilization method, post-treatment semen volume, and post-treatment semen density. Subsequent multivariate logistic regression analysis showed that female age, normal fertilization, day 2 cleavage-stage embryo grade, day 3 cleavage-stage embryo grade, and fertilization method were significant factors affecting the formation of viable blastocysts in DOR patients. To enhance the applicability of this model in clinical practice, the study included a nomogram for the identified factors.

This study indicates that age is a risk factor for the formation of viable blastocysts in patients with diminished ovarian reserve (DOR). La Marca A [14] suggests that age is a significant determinant affecting the outcomes of assisted reproductive technologies (ART), primarily because increasing age directly leads to a decline in ovarian reserve function and oocyte quality. Additionally, structural and functional abnormalities in the oocyte’s spindle apparatus and mitochondria may result in atypical cell division, consequently increasing the rate of chromosomal abnormalities in embryos. Research by Soler A et al. [15] reveals that patients over 35 years of age have approximately a 25% higher rate of embryonic chromosomal abnormalities compared to those 35 years old or younger, with a significant correlation between chromosomal abnormality rates and blastocyst formation. Furthermore, studies by Ezoe K et al. [16] indicate that with the advancing age of both partners, the incidence of embryos with 3 pronuclei (3PN) also rises. Compared to embryos derived from 2 pronuclei (2PN), those from 1 and 3PN sources exhibit a significantly reduced rate of blastocyst formation. This aligns with our study’s findings, where normal fertilization is a favorable factor for the formation of viable blastocysts.

This study suggests that intracytoplasmic sperm injection (ICSI) is a risk factor for viable blastocyst formation in patients with diminished ovarian reserve (DOR). Due to the invasive nature of ICSI, its impact on embryo development and blastocyst formation remains a subject of debate. Research by Van Landuyt L et al. does not associate the method of fertilization with differences in embryo development and blastocyst formation [17]. However, studies by Yin H et al. indicate that the rates of blastocyst formation and high-quality blastocyst development are higher in in vitro fertilization (IVF) cycles compared to ICSI cycles. This may be related to the inherent impact of the ICSI technique on the developmental potential of embryos [18]. Firstly, during the IVF process, the zona pellucida of the oocyte exerts a relative selection for sperm during the natural sperm–oocyte binding process. ICSI bypasses this selective step, potentially allowing for the fertilization of oocytes by morphologically normal but compromised sperm, which could further affect embryo development [19]. Additionally, ICSI necessitates the injection of a certain amount of exogenous substances due to the requirement of sperm immobilization, potentially influencing the developmental potential and safety of the offspring [20]. Secondly, the ICSI technique increases the duration of extra-embryonic manipulations compared to conventional IVF, due to the needs for denudation and microinjection. The consequent changes in temperature and osmotic pressure may directly affect the embryo’s developmental potential. Thirdly, ICSI demands high technical proficiency from the embryologist. There is a risk of damage to the aster, spindle, microtubules, and microfilaments due to operator factors, which could lead to abnormal embryo division, or even degeneration, severely compromising the developmental potential of the embryo [21].

This study reveals that the day 2 cleavage-stage embryo grading and day 3 cleavage-stage embryo grading are influential factors for the formation of viable blastocysts in patients with diminished ovarian reserve (DOR). The grading of D2 and D3 cleavage-stage embryos in this study is based on the Istanbul consensus criteria published by ESHRE in 2011 [22], while the blastocyst grading refers to the Gardner blastocyst scoring system [23]. Research by Wong et al. suggests that combining time-lapse imaging analysis with gene expression profiling to evaluate day 2 embryos can predict embryo development and blastocyst formation [24]. Significant correlations exist between the quality of day 3 cleavage-stage embryos and blastocyst formation. Studies have shown that when the number of blastomeres on day 3 is fewer than five, the developmental potential of the embryo is reduced due to delayed development, subsequently affecting blastocyst formation [25]. High-quality day 3 embryos with 7–9 blastomeres, low fragmentation, normal cell size, good uniformity, and appropriate developmental stage and without abnormalities such as multinucleation, smooth endoplasmic reticulum, and vacuolization have a higher potential to develop into blastocysts. This aligns with our study’s findings that day 3 embryo grading is a determinant factor for blastocyst formation. Awadalla M’s research suggests that day 3 cleavage-stage embryos with eight blastomeres and less than 10% fragmentation are more likely to form blastocysts and have higher live birth rates [26]. However, embryologists’ assessments of any embryonic structures are subject to subjective factors, resulting in varying degrees of inter-observer differences. Eastick J. proposes that the introduction of time-lapse systems into laboratories allows for continuous monitoring of developing embryos, enabling the observation and discovery of dynamic structures, such as cytoplasmic strings, which could be a crucial information for predicting the developmental potential of embryos [27].

Achieving successful pregnancy in patients with diminished ovarian reserve (DOR) is a particularly challenging problem in the field of assisted reproductive technologies (ART). Currently, a wealth of research is exploring various techniques to improve the quality of oocytes and increase the number of oocytes in DOR patients, including mitochondrial transfer, activation of primordial follicles, in vitro culture of follicles, and the regeneration of oocytes from various stem cells [28]. Given the precious nature of embryos in DOR patients, guiding them on how to make optimal use of the obtained embryos is an urgent clinical issue that needs to be addressed. This study identified five factors impacting the formation of viable blastocysts in DOR patients: female age, normal fertilization status, day 2 cleavage-stage embryo grading, day 3 cleavage-stage embryo grading, and fertilization method. A predictive model based on these factors, represented by a nomogram, demonstrates good clinical predictive value and efficacy for clinical interventions and personalized medicine in DOR patients. However, this study has certain limitations. First, it is a single-center, retrospective study with a relatively limited sample size, lacking external validation from multicenter trials and prospective studies with larger cohorts. Second, due to the numerous uncertain factors affecting blastocyst formation and the scarcity of clinical models for viable blastocyst formation in DOR patients, there is a lack of horizontal comparison between the strengths and weaknesses of other models.

## Data Availability

Not applicable.
